# Effect of Climate Change on Soil Temperature in Swedish Boreal Forests

**DOI:** 10.1371/journal.pone.0093957

**Published:** 2014-04-18

**Authors:** Gunnar Jungqvist, Stephen K. Oni, Claudia Teutschbein, Martyn N. Futter

**Affiliations:** 1 Department of Earth Sciences, Uppsala University, Uppsala, Sweden; 2 Department of Aquatic Sciences and Assessment, Swedish University of Agricultural Sciences, Uppsala, Sweden; University of Missouri, United States of America

## Abstract

Complex non-linear relationships exist between air and soil temperature responses to climate change. Despite its influence on hydrological and biogeochemical processes, soil temperature has received less attention in climate impact studies. Here we present and apply an empirical soil temperature model to four forest sites along a climatic gradient of Sweden. Future air and soil temperature were projected using an ensemble of regional climate models. Annual average air and soil temperatures were projected to increase, but complex dynamics were projected on a seasonal scale. Future changes in winter soil temperature were strongly dependent on projected snow cover. At the northernmost site, winter soil temperatures changed very little due to insulating effects of snow cover but southern sites with little or no snow cover showed the largest projected winter soil warming. Projected soil warming was greatest in the spring (up to 4°C) in the north, suggesting earlier snowmelt, extension of growing season length and possible northward shifts in the boreal biome. This showed that the projected effects of climate change on soil temperature in snow dominated regions are complex and general assumptions of future soil temperature responses to climate change based on air temperature alone are inadequate and should be avoided in boreal regions.

## Introduction

There is increasing consensus that the global climate is getting warmer in comparison to pre-industrialized times [1], [2]. This is supported by a growing number of observations; changing weather patterns [3], increasing water temperatures [4], [5], rising sea levels [6], melting of ice and snow in the arctic or subarctic regions [7], [8] amidst other signals. The effects of global warming are not restricted to air temperature alone but can also change precipitation patterns as well as soil temperature. Snow is a very effective insulator and snow cover can effectively decouple air and soil temperature during the winter. Thus, soil temperature response to climate change may differ from that of air temperature depending on changes in the timing and duration of the winter snowpack [9].

Despite their importance in controlling watershed biogeochemical and hydrological processes, soil temperature data are generally less available than air temperature measurements. Soil temperature controls biogeochemical processes such as dissolved organic carbon export [10], length of growing season [11], [12], rates of mineralization [13], [14] or decomposition of soil organic matter [15] and nutrient assimilation by plants [16], [17], weathering of base cations [18] as well as forest productivity [19].

Soil temperature is influenced by many factors. The insulating effects of snow as well as changes in the timing and intensity of snowfall can have significant feedback effects on soil temperature dynamics in the northern boreal landscape during winter and spring [20]. Studies have shown the possibility of colder soils in a warmer future in high latitude sites [21] due to increasing soil frost depth associated with reduced snow cover or faster melt rates [22], [23]. Winter soil frost could result in more fine root mortality [24] and intensify leakage of soil nutrients [25]. The increasing intensity of freeze-thaw cycles [26] could affect overall soil aggregate stability [27] and/or hydrological processes and flow paths as ice fills up the soil pore spaces [28]. The biogeochemical effects of intensified freeze-thaw cycles could include altered rates of microbial respiration, cell wall lyses and changes in the rate of decomposition of soil organic matter [29]. The latter could lead to increasing CO_2_ emissions from soils [13], [30] resulting in a positive feedback to climate change [15]. The relative stability of carbon stored in high latitude forest catchments could be altered [31], thus shifting the boreal forest from sink to a source of carbon as temperature changes [32].

Therefore, there is an urgent need to properly represent soil temperature dynamics in forest carbon cycle models so as to constrain the uncertainty in future projections of environmental response to climate change. Here we present an extended version of the soil temperature model [33] used in the INCA suite of integrated catchment models [34]–. The objective of this study is to address the question of how future climate change could affect soil temperature response in a set of well-monitored forest sites aligned in a south-north gradient in Sweden. Given the availability of long term soil temperature measurements at a series of depths in the four Swedish Integrated Monitoring (IM) sites, we hope that this study will lead to improved soil temperature simulations when thermal conductivity is allowed to vary throughout the soil profile.

### Ethics Statement

Soil temperature measurements were collected at the four Swedish Integrated Monitoring sites as part of the routine long-term monitoring conducted by the Swedish University of Agricultural Sciences, IVL, and others [38]. All sites have public access so no additional permissions were required. Our field studies did not involve endangered or protected species. All soil temperature data are publicly available through the Integrated Monitoring Program web site hosted by the Swedish University of Agricultural Sciences (http://webstar.vatten.slu.se/db.html)

## Study Area

**Figure 1:**
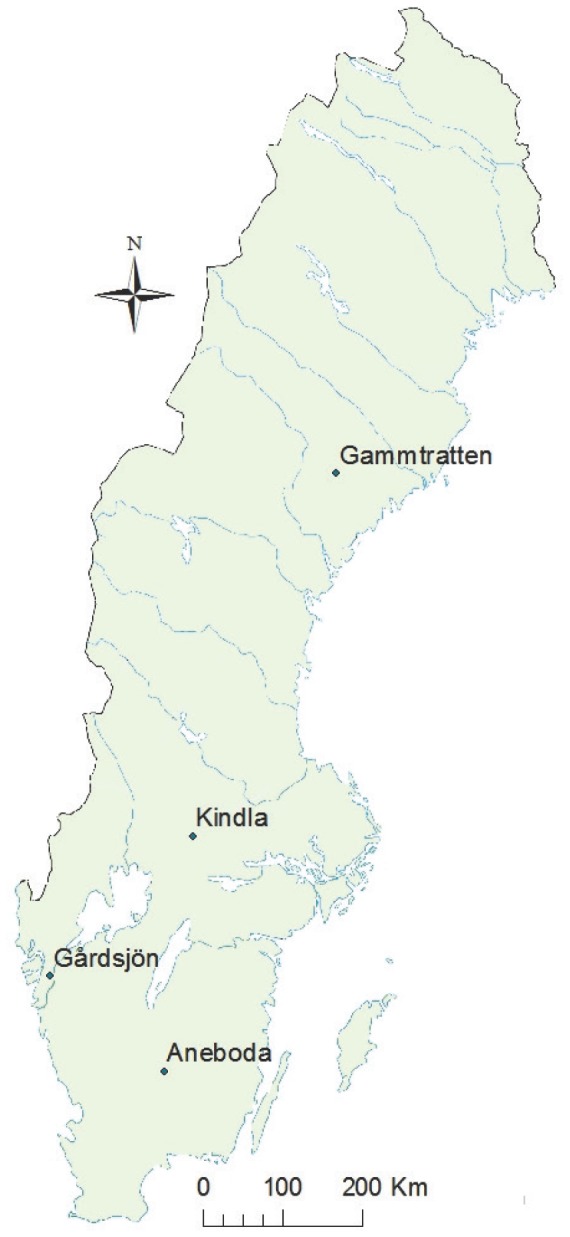
Map of study catchments as aligned along the climatic gradient of Sweden.

This study was conducted at the Swedish Integrated Monitoring sites (IM); Aneboda, Gårdsjön, Kindla and Gammtratten (Figure 1). The Swedish IM sites are a set of well monitored headwater forest catchments and are part of a Europe-wide initiative to collect long time series of data on a number of key ecosystem variables. Data from the IM sites are used to study long term changes in fundamental catchment processes across the climatic gradient of Sweden. Due to the lack of land management such as agriculture or forestry, well-defined catchment boundaries and the availability of long time series of climate data, the IM catchments are suitable for the modeling exercise presented in this study.

### 2.1 Specific catchment descriptions

The Aneboda catchment (0.19 km^2^) is situated in the Småländska highlands (57°07′N, 14°03′E) and the biome is characterized as Boreo-nemoral (Figure 1). The mean air temperature in the catchment was 6.5°C and precipitation was 796 mm/yr (1996–2008). Norway spruce (*Picea abies*) is the dominant tree species (73%) with some Birch (*Betula* spp.) (20%). Other tree species with minor representation in the catchment include Scots Pine (*Pinus sylvestris*) (3%), Beech (*Fagus sylvatica*) (1%) and Alder (*Alnus* spp.) (2%). The soil is dominated by glacial till, with the proportion of wet soils amounting to 17% on granite bedrock. In 2005, the catchment was hit by a severe storm followed by a bark beetle infestation that caused extensive damage to the forest [38].

The Gårdsjön catchment (58°03′N, 12°01′E) is located in Bohuslän in the Swedish west cost approximately 10 km from the sea (Figure 1). The catchment (0.04 km^2^) is the smallest of all the four IM catchments. The catchment biome is also characterized as Boreo-nemoral with mean annual air temperature of 7°C and annual precipitation of 1111 mm/yr (1996–2008). The vegetation cover is dominated by Norway spruce (65%) but Birch (14%) and Scots Pine (17%) are also present. The catchment geology also consists of granitic bedrock underlying glacial till soils. Soils in Gårdsjön are very shallow and interrupted by bedrock outcrops in some part of the catchment. The proportion of wet soils within the catchment is 10%.

Kindla is situated towards the middle of Sweden (59°45′N, 14°54′E) and has an area of about 0.2 km^2^ (Figure 1). The catchment biome is characterized as Southern-boreal with mean air temperature of 5.2°C and mean annual precipitation of 854 mm/yr. The catchment also has significant bedrock outcrops (41%) and is quite steep with elevation ranging from 100–400 meters above sea level (m a.s.l). The vegetation is dominated by Norway spruce (83%) but Birch (14%) and Scots Pine (2%) are found in the catchment. The soil consists of 24% wet soil with mire in the center of the catchment.

Gammtratten is the northernmost (63°51′N, 18°08′E) and largest (0.4 km^2^) of all the IM catchments (Figure 1) with mean air temperature of 2.4°C and annual precipitation of 579 mm/yr. The catchment is categorized as middle-boreal. Vegetation mainly consists of Norway spruce (70%), with some Birch (16%) and Scots Pine (13%). Pine is mostly found in the higher elevations of the catchment, where there are several small mires. The percentage of wet soils was 16% with presence of granite bedrock underlying the glacial till soil.

## Climate

### 3.1 Historical climate

The data requirements for the soil temperature modelling and analysis presented here include observed average daily air and soil temperature. Continuous time series of both air temperature and precipitation (1996–2008) for each IM site were obtained from the IM database at Swedish University of Agricultural Science. Future projections in Gammtratten were driven by climate data from the nearby Krycklan catchment [39]. Our assumption of using Krycklan was justified as both catchments have similar weather patterns (with R^2^ of 0.73 for precipitation and 0.98 for air temperature). Due to large gaps in the soil temperature data, depths with the most consistent long term series were used for model calibration and validation in each catchment. Soil temperatures at three depths (Table 1) were modelled at each site.

**Table 1:** Catchments and their soil profile depths used in the modelling analysis presented in this study.

Catchment	Depth in soil profile (cm)
Aneboda, top layer	10
Aneboda, middle layer	32
Aneboda, bottom layer	58
Gårdsjön, top layer	0*
Gårdsjön, middle layer	10
Gårdsjön, bottom layer	25
Kindla, top layer	5
Kindla, middle layer	20
Kindla, bottom layer	35
Gammtratten, top layer	5
Gammtratten, middle layer	29
Gammtratten, bottom layer	40

* Simulated as 1 cm depth.

### 3.2 Future climate

#### 3.2.1 Climate models

Future climate data were based on ENSEMBLES project outputs [40]. The ENSEMBLES project utilized an ensemble of Regional Climate Models (RCMs) driven by different Global Climate Models (GCMs), to generate a matrix of possible climate projections under International Panel on Climate Change (IPCC) emission scenarios [1]. The ENSEMBLES project generated a range of possible climate outcomes which help to make future projections more statistically reliable. Since the climatic factors (e.g. air temperature, winds, etc.) are a connected system in the atmosphere and extend all over the world, the GCMs operate at a global scale. The emission scenarios in the GCMs were based on assumptions about future population, economic development, etc. These assumptions were then translated into anthropogenic emission scenarios used as forcing for the GCMs, though different feedbacks forcing are also integrated include melting of ice caps [23] and changes in soil CO_2_ balances [12] amongst others. As a result, all GCMs are designed to model the complex climatological system of the earth with each GCM having certain processes and feedbacks represented differently. This leads to differences between GCM representations of the future as a result of varied responses to forcing and differences in RCM responses even at much finer resolutions.

#### 3.2.2 Bias correction of climate models

Downscaling is often used in climate impact studies to create outputs with higher resolution [41]–[43]. Despite their finer resolution, RCMs are still coarse for direct applications in local impact studies as there are often biases between RCMs outputs and measured site specific conditions. Therefore we utilized distribution mapping in this study to correct biases in RCM-simulated temperature and precipitation. This approach has been shown to be the best bias correction method for small and meso-scale catchments in Sweden [44], [45]. Here, 15 RCM ensembles were bias corrected to local IM conditions for the period 2061–2090 (Table 2). The general principle of distribution mapping is to fit cumulative distribution functions (CDFs) of observed data to CDFs of RCM outputs in the control period (1996–2008) according to Eqn. 1.
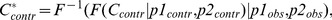
(1)where *C* is the climate variable of interest (precipitation or temperature), C* represents the bias-corrected climate variable, *F* stands for the theoretical CDF (Gamma or Gaussian) and *F^−1^* for its inverse, *p1* and *p2* are the distribution parameters (*α* and *β* for Gamma distribution, *μ* and σ for Gaussian distribution), the subscripted expression *obs* indicates observations and *contr* stands for the RCM-simulated control run (1996–2008).

**Table 2:** Underlying RCMs for the bias corrected site-specific climate scenarios, their notation, resolutions, driving GCMs, emission scenarios and performing institutes.

No.	Notation	Institute	RCM	Resolution	Driving GCM	Emission scenario
1	C41_HAD	C4I	RCA3	25 km	HadCM3Q16	A1B
2	DMI_ARP	DMI	HIRHAM5	25 km	ARPEGE	A1B
3	DMI_BXM	DMI	HIRHAM5	25 km	BCM	A1B
4	DMI_ECH	DMI	HIRHAM5	25 km	ECHAM5	A1B
5	ETHZ	ETHZ	CLM	25 km	HadCM3Q0	A1B
6	HC_HAD0	HC	HadRM3Q0	25 km	HadCM3Q0	A1B
7	HC_HAD3	HC	HadRM3Q16	25 km	HadCM3Q16	A1B
8	HC_HAD16	HC	HadRM3Q3	25 km	HadCM3Q3	A1B
9	KNMI	KNMI	RACMO	25 km	ECHAM5	A1B
10	MPI	MPI	REMO	25 km	ECHAM5	A1B
11	SMHI_BCM	SMHI	RCA	25 km	BCM	A1B
12	SMHI_ECH	SMHI	RCA	25 km	ECHAM5	A1B
13	SMHI_HAD	SMHI	RCA	25 km	HadCM3Q3	A1B
14	CNRM	CNRM	Aladin	25 km	ARPEGE	A1B
15	ICTP	ICTP	RegCM	25 km	ECHAM5	A1B

Note that both the numbering sequence and/notations are used interchangeably to refer to each RCM throughout this study.

The derived distribution parameters from Eqn. 1 were then applied to future series to adjust the RCM climate variables (C_scen_) according to Eqn 2. The subscripted expression *scen* indicates the RCM-simulated scenario run (2061–2090). The bias corrected ensemble RCM data were used as driving variables for the prediction of future soil temperatures at all depths in our study catchments except Gammtratten which was driven by bias corrected series from nearby Krycklan catchment. We refer readers to *Teutschbein and Seibert* [44] for more detailed descriptions of this technique.
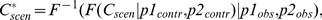
(2)


## Modelling Analyses

### 4.1 Background on soil physics

The soil temperature model was derived from two differential equations describing combined water and heat flows in seasonally frozen soil [46]. These equations were derived from the law of conservation of energy and mass (Eqns. 3 and 4) and the fact that water and heat spread out in the soil profiles along gradients of water potential and temperature (Darcy and Fourier laws).
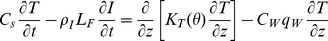
(3)

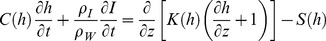
(4)where *Z* (m) is a space coordinate, *T *(°C) is soil temperature, *K_T_* (Wm^−3^°C^−1^) is the soil thermal conductivity, *L_F_* (J kg^−1^) is latent heat of fusion and water, *C_S_* (J m^−3^°C^−1^) is volumetric specific heat of the soil, 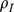 (kg m^−3^) is the density of ice, 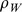 (kg m^−3^) is the density of water, 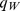 (m s^−1^) is flow of water, *q* (dimensionless) is a volumetric water content, *I* (dimensionless) is a volumetric ice content, *h* (m) is the soil water potential, *C(h)* (m^−1^) is the differential moisture capacity, *K(h)* (m s^−1^) is the unsaturated hydraulic conductivity of the soil matrix and *S(h)* (dimensionless) represents the water taken up by the roots. These equations calculate water and heat flow based on soil properties; the water retention curve, unsaturated and saturated hydraulic conductivity, heat capacity (including latent part during thawing/melting) and thermal conductivity [33].

#### 4.2 Rankinen model and the extended version

The model used in this study was based on the soil temperature model described in *Rankinen et al.* [33] which was based on the simplifications of Eqns. (3) and (4). The model calculates soil temperature at different depths at a daily time step with the full consideration of the influence of snow cover. Simplifications made by *Rankinen et al.* [33] neglected the influence of changes in soil water content on soil temperature. This made it easier to solve Eqn. (4) and all water related terms from Eqn. (3) with the exception of the ice term.

In evaluating the derivatives from Eqn. (3), boundary conditions were set as *T_SURF_* (temperature at the surface, replaced by air temperature *T_AIR_* in Eqn. (5), *T_Z_* (temperature in the soil evaluated according to space reference *Z*) and *T_l_* which is soil temperature at *2Z_S_*. For purposes of simplification, this last term was assumed to equal *T_Z_*, indicating that heat flow under the soil layer of interest could be taken into consideration. The derivative of the soil thermal conductivity in the soil profile was replaced by a constant, representing the average thermal conductivity of the soil. However, the soil thermal conductivity was linked with the soil moisture [46], which varies throughout the soil profile and throughout the seasons. As a result of this simplification, the model lost its validity under very wet or dry conditions but greatly simplified estimation of soil temperature (Eqn. 5).
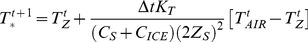
(5)As eqn. (5) did not take the influence of snow into account, the equation was extended by an empirical relationship (Eqn. 6) that could simulate the insulating the effect of snow cover and soil temperature could thus be calculated (Eqn. 7).
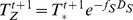
(6)

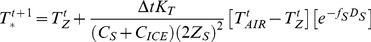
(7)where *K_T_* (Wm^−1^°C^−1^) is the thermal conductivity, *C_S_* (J m^−3^°C^−1^) is the specific heat capacity of the soil, *C_ICE_* (J m^−3^°C^−1^) is the specific heat capacity due to freezing and thawing (latent part) as well as *f_s_* (m^−1^) which represents an empirical snow parameter. These represent model parameters that can be used in the model calibration. *T_AIR_* (°C) and *D_S_* (*mm*) represent air temperature and snow depth.

This study further explored the possibilities of improving the soil temperature simulations (Eqn. 8) when the thermal conductivity is allowed to vary throughout the profile. This extended version also utilized temperature inputs below the soil layer of consideration. Therefore, parameters controlling the lower soil thermal conductivity *K_T,LOW_*, lower soil specific heat capacity *C_S,LOW_*, and lower soil temperature *T_LOW_* were also added to the model (Eqn. 8).
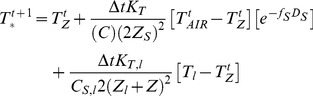
(8)where *Z_l_* is a constant indicating the space coordinate for the lower temperature influence. 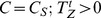 or 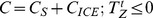


The model was implemented in MATLAB.

### 4.3 Model simulations and projections

**Figure 2:**
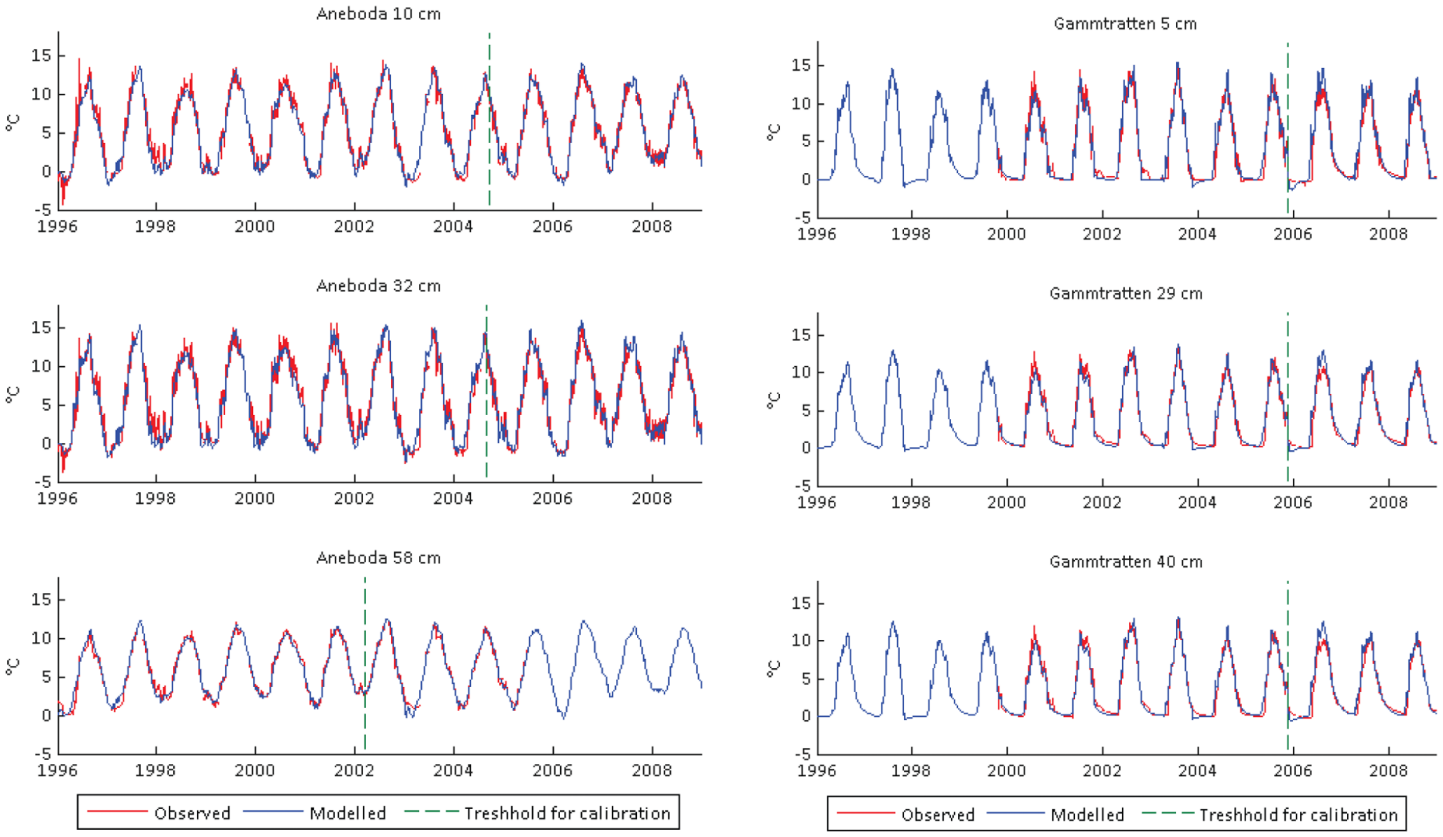
Simulated and observed soil temperatures for the representative southern (Aneboda) and northernmost (Gammtratten) catchments.

The model was calibrated to present day condition (1996–2008). Since there were gaps in soil temperature observations, the calibration and validation periods were not split by a specific date but by examining the number of observations available and then splitting the data series in thirds. The first two thirds of the series were used for calibration, making the calibration and validation periods differ between catchments and soil depths (e.g. Figure 2). A Monte Carlo sampling technique was used for model optimization by random sampling of parameter spaces as well as tracing out the structure of the model output [47]. This allows proper evaluation of multiple combinations of parameter settings in models with significant uncertainty in inputs and outputs. By changing one parameter at a time (as is usually done in manual calibration), model performance might be less than optimal as there would only be one degree of freedom. However, evaluating multiple combinations of parameters manually is often impossible in more complex systems.

Parameters that have a physical interpretation (grey box modeling) must be combined with prior knowledge of the system. In the soil temperature model described in this study, six out of the seven parameters (Table 3) have clear physical interpretations as their values are limited by their physical boundaries. It is therefore of importance that the model parameters are set within a reasonable range when performing Monte Carlo analysis (Table 3). Choosing the parameter range for individual sites and depths was difficult. However, there are a number of strategies in choosing a suitable parameter ranges. These include literature values, experimental results and measurements as well as expert judgments. The range at which the upper soil temperature parameters (*C_S_*, *K_T_*, *f_S_* and *C_ICE_*) were allowed to vary was based on the parameter range proposed by [33], [48]. The range of the lower soil temperature parameters (*T_LOW_*, *C_S,LOW_* and *K_T,LOW_*) were unknown, and were therefore set according to judgments.

**Table 3:** Parameter ranges for the Monte Carlo simulations.

Parameter	Unit	Monte Carlo ranges
Specific heat capacity of soil, *C_S_*	J m^−3^°C^−1^	0.5–3.5 (10^6^)
Soil thermal conductivity, *K_T_*	Wm^−1^°C^−1^	0–1
Specific heat capacity due to freezing and thawing, *C_ICE_*	J m^−3^°C^−1^	4–15 (10^6^)
Empirical snow parameter, *f_S_*	m^−1^	0–10
Lower temperature, *T_LOW_*	°C	0–1
Thermal conductivity, lower part, *K_T,LOW_*	Wm^−1^°C^−1^	0–1
Specific heat capacity of soil, lower part, *C_S,LOW_*	J m^−3^°C^−1^	0.5–3.5 (10^6^)

**Figure 3:**
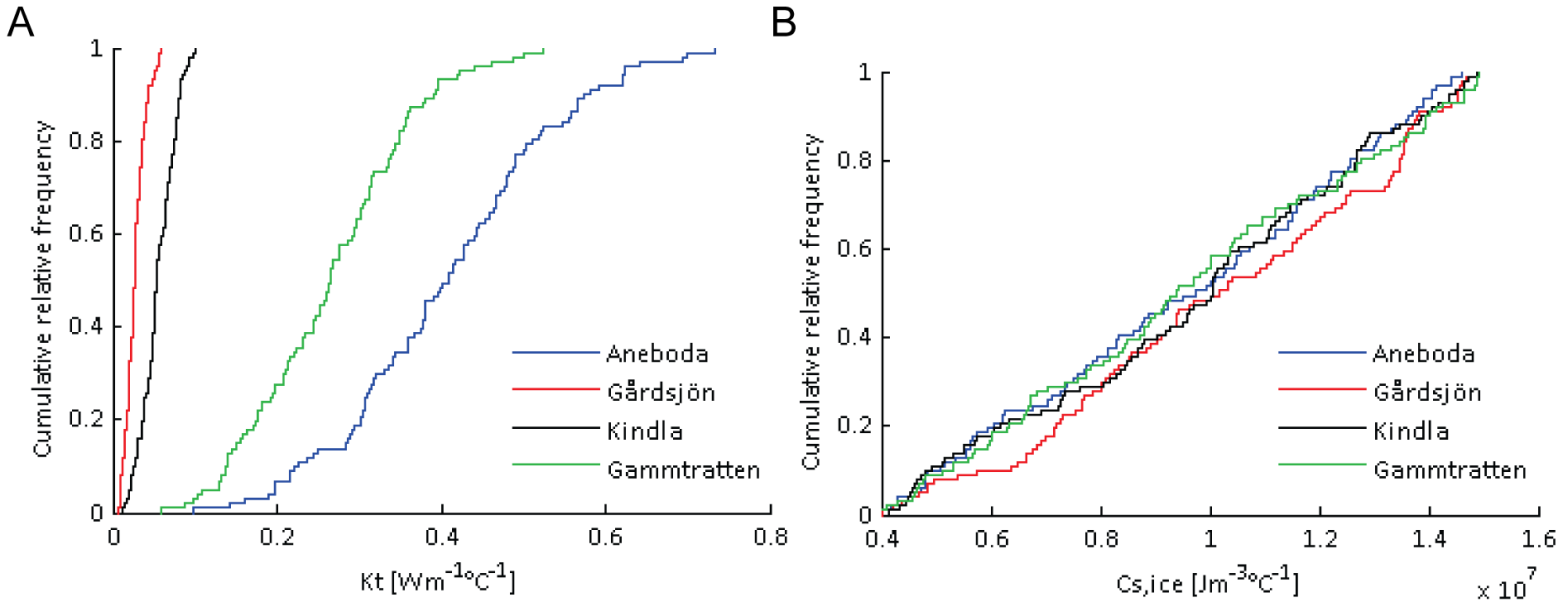
Cumulative relative distributions for *K_T_* (plot a) and *C_S,ICE_* (plot b) from the Monte Carlo simulations of middle soil layers across the four IM catchments.

The Monte Carlo analysis was conducted by running the model 100 000 times, sampling a new set of randomized parameters for each model run from the chosen parameter ranges. This process was repeated for each site and for all depths. The high number of simulations increased the likelihood that the whole parameter range was well sampled. Each parameter was sampled using the MATLAB rand() command, scaled to fit each parameter range. Model performances during calibration period were measured using the Nash and Sutcliffe (NS) statistic [49] while validation period performance was measured using a combination of NS, R^2^ (calculated based on linear fitting of observed and modeled values) and root mean square error (RSME). The Monte Carlo outputs were further analyzed using cumulative distribution frequency (CDF) of the top 5000 simulations. A sensitive parameter tends to have a non-uniform posterior distribution while a non-sensitive parameter distribution spreads over the entire range. Parameter values for the behavioral runs (top 100) and bias-corrected climate data were used for projecting future conditions (Figure 3). The projected series were further analyzed on both annual and seasonal scales for each of the 15 RCMs during 2061–2090.

## Results

### 5.1 Soil temperature simulations

The model successfully captured inter-annual variation in soil temperature at all catchments. Figure 2 shows the model calibration in Aneboda and Gammtratten to illustrate the south-north gradient. Model performance in Aneboda ranges from NS value of 0.96–0.97 (calibration) and 0.96–0.97 (validation). The model also performed well in Gårdsjön (NS 0.97–0.98), Kindla (NS 0.95–0.97) and Gammtratten (NS values of 0.96–0.98). There was greater variability in upper and middle layer soil temperatures at Aneboda than at Gårdsjön. The variability increased northward and was consistent in the upper soil layer with the increasing influence of air temperature. The model failed to capture some of the winter soil temperature dynamics (Figure 2) at Gammtratten, making this catchment a unique site with clear insulating effects of snow cover as the soil temperature flattens out during winter months (Figure 2).

### 5.2 Uncertainty analysis

The uncertainty analysis revealed parameter equifinality. This means that there is no single best model parameter set and that many model state descriptions can generate equally good calibration outputs [50]. For example, parameter *C_S,ICE_* was highly insensitive (Figure 3), making it hard to identify optimum values of this parameter to best represent present day soil conditions. Additionally, it was hard to make cross-catchment comparisons since soil temperatures were simulated at different depths for the different sites. However, K*_T_* was clearly the most sensitive parameter that differentiates each catchment along a south-north gradient in Sweden (Figure 3). Both Kindla and Gårdsjön have narrower K_T_ ranges compared to Aneboda and Gammtratten. This shows the importance of soil thermal conductivity in regulating soil temperature in high latitude catchments.

### 5.3 Ensemble projections

#### 5.3.1 Changes in air temperature

**Figure 4:**
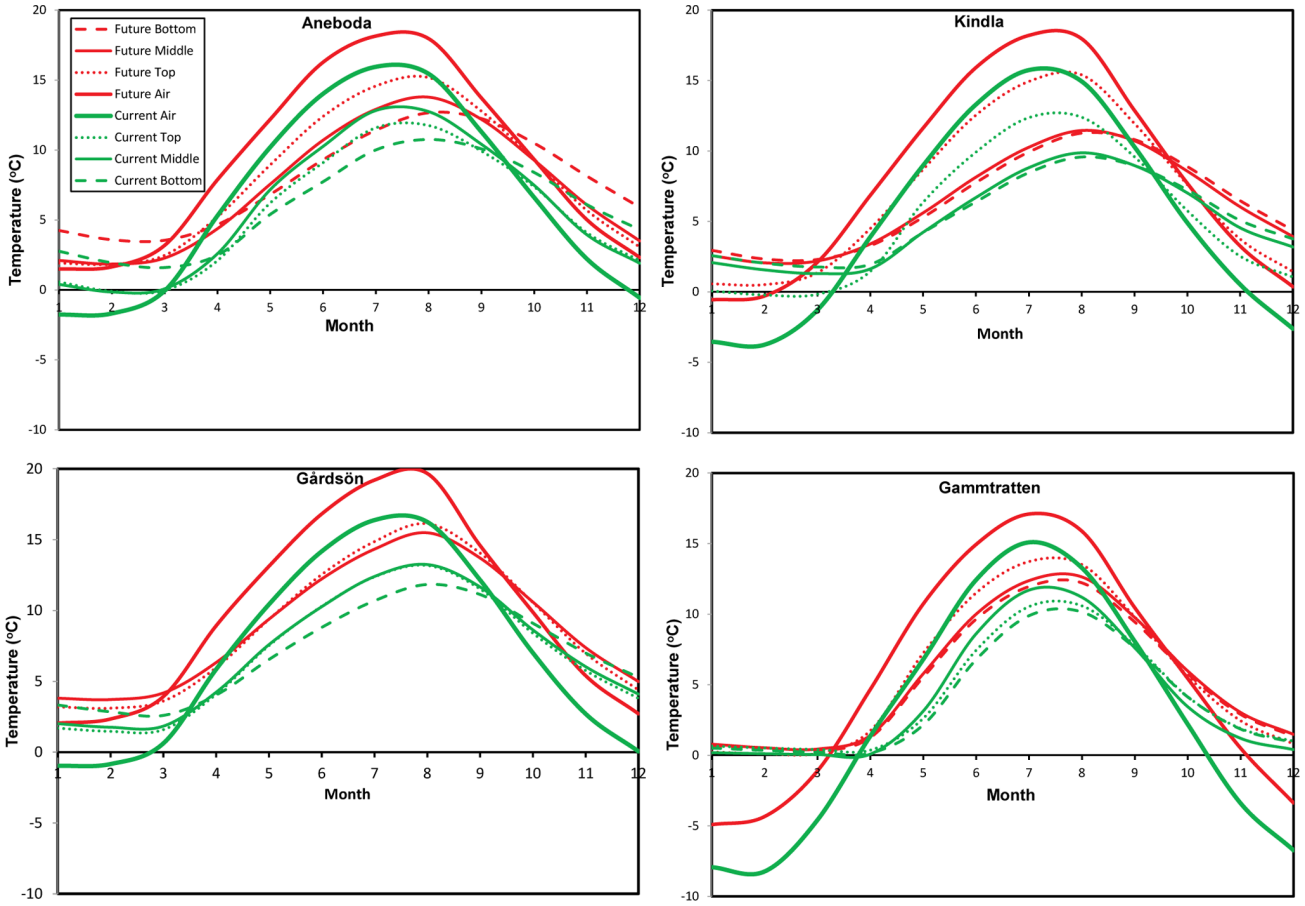
Ensemble projection of air versus soil temperature across the soil profile in the four Integrated Monitoring catchments.

**Figure 5:**
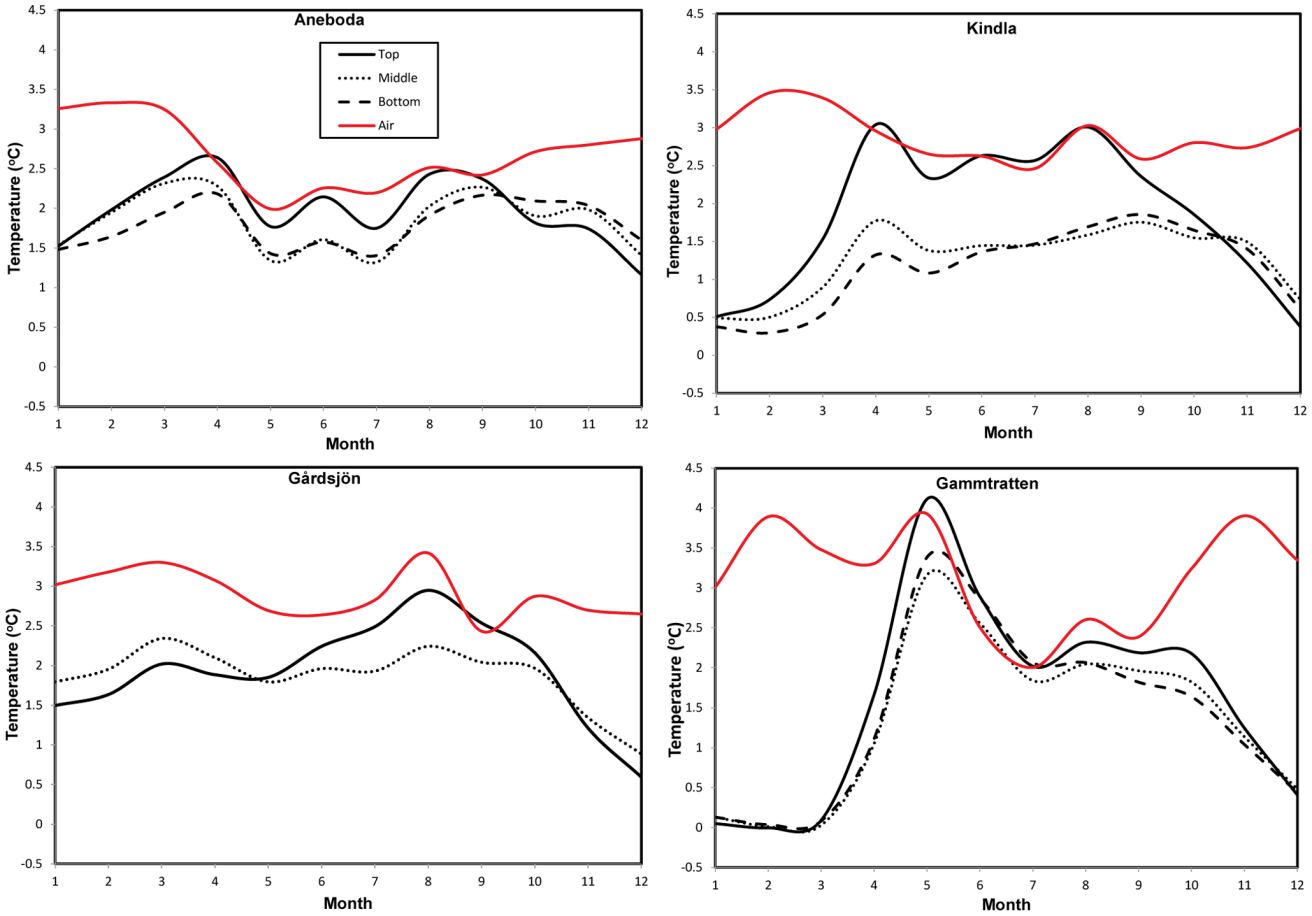
Corresponding change in the future air and soil temperature relative to the current conditions (shown in Figure 4).

The RCM ensemble projected a range of possible future climates across Sweden (Figure 4). Changes in air temperature are projected to be greatest in the colder months (November–April) with the magnitude of change increasing northward. Future air temperatures <0°C were projected to decrease markedly in the northern catchments with possibility of complete disappearance of winter air temperatures <0°C in the southern catchments (Figure 4). The largest change in air temperature would be observed in winter (about 3°C rise) with a possibility of autumn warming up to 2.5°C (Figure 5). However, Gårdsjön showed the possibility of sporadic warming up to 3.4°C in August, thereby shifting the warmest month of the year away from July in that catchment. The patterns of future change in air temperature in Kindla are fairly similar to the patterns projected in Gårdsjön (Figure 5). There could also be an abrupt rise in air temperature up to 3°C in the month of August with the biggest winter air temperature change 3°C.

The northernmost catchment showed markedly different magnitude and patterns of change compared to the rest of the southern catchments. Ensemble projections showed that winter air temperature change could be largest (3.4°C rise) in Gammtratten region with a possibility of sporadic warming events up to 3.9°C in February and May. Autumn warming, though slightly visible in other catchments, could become more common in this region in the future (up to 3°C rise). However, there could also be sporadic warming events peaking in November (3.9°C rise) before the onset of the colder winter months (Figure 5). These results show the possibility of increased variability and fluctuations of future climate across Sweden.

#### 5.3.2 Changes in soil temperature

**Figure 6:**
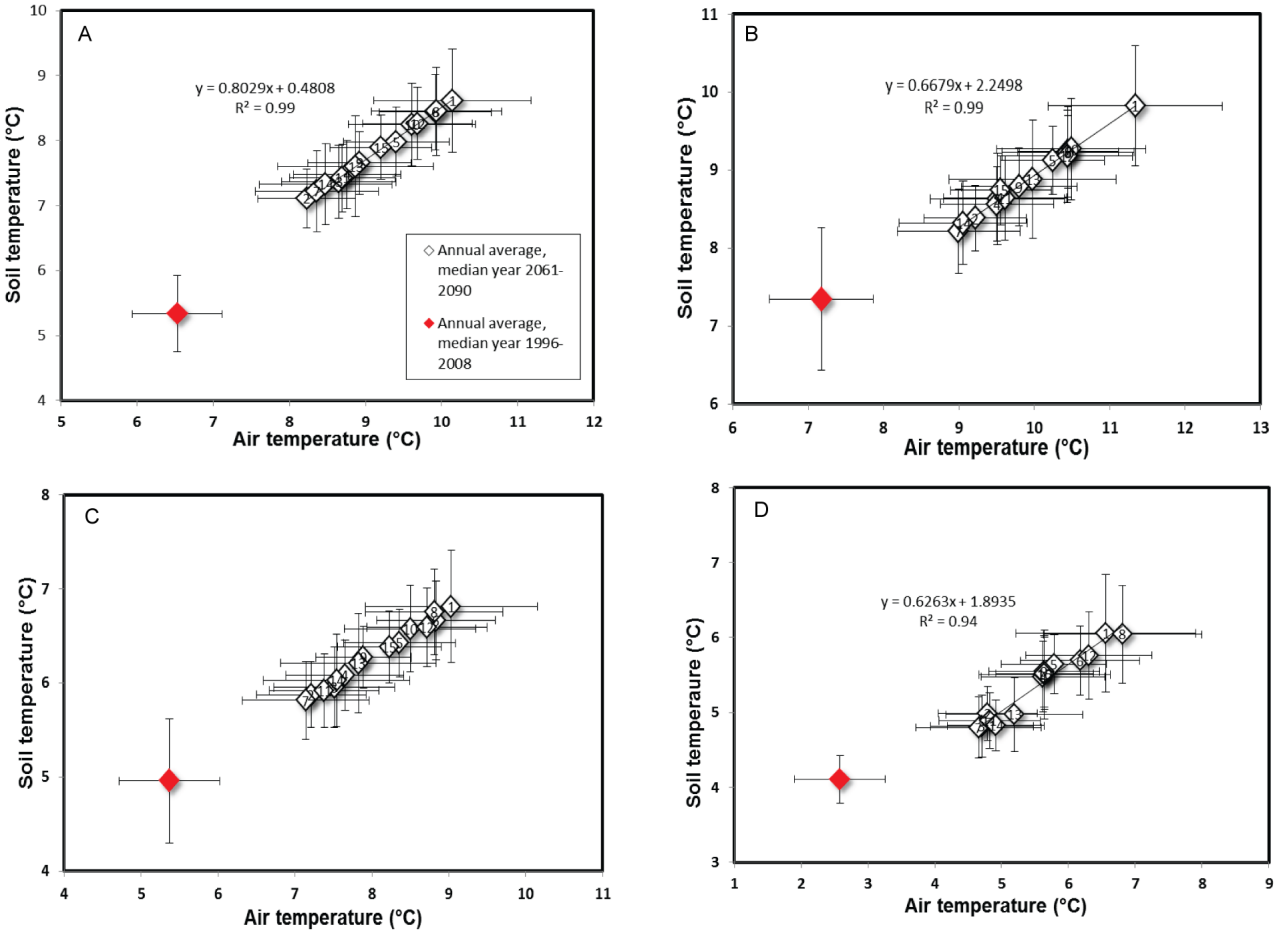
Mean annual mean air-soil temperature for the control period (calibration/validation period) using median year (*red diamond*) versus mean annual air-soil temperature ensembles (*plain diamonds*. Label 1–15 denote each RCM and fully described in Table 2), using ensemble median for Aneboda (A), Gårdsjön (B), Kindla (C) and Gammtratten (D) middle soil layer. *Error bars* represent corresponding annual standard deviations.

The overall ensemble showed markedly different winter soil temperature responses to future climate change across the soil profile in each catchment (Figure 4). Though soil temperature responses increase northward, the pattern of change varies significantly across months in each catchment (Figure 5). Despite their southern location, patterns of soil temperature response in Gårdsjön are different from Aneboda. Changes in soil temperatures in Gårdsjön were less variable and more synchronous with air temperature (Figure 5). Although air temperature could also be changing more rapidly than soil temperatures in this catchment on an annual scale (Figure 6), the largest change was projected to occur in the top soil with differences between the top/lower layers responses being most pronounced in late summer and early autumn.

While the two southern catchments had relatively moderate soil temperatures throughout the year, changes could be more pronounced in Kindla and Gammtratten sites, the more northern sites (Figure 5). The biggest soil temperature changes in Kindla were projected to occur in April–August and the effect was most pronounced in the topmost layer relative to the middle and bottom layers which showed fairly similar response patterns. Middle and bottom soil temperatures could rise by about 1.5°C (about 2.5°C for topmost layer) in the future for most months of the year, except December–February where the model projected a temperature of about 0.5°C.

Despite the largest air temperature changes in the northernmost Gammtratten catchment, there was no corresponding soil temperature responses in the winter unlike other catchments where soil could get warmer during the cold and dormant winter season. This clearly shows the insulating effects of extensive snow cover in this region. In contrast to the almost unchanging in soil temperature in winter months, there was a steady rise in soil temperature after March and this could reach up to 4°C in May in Gammtratten (the largest change in all catchments). The subsequent stabilization in the summer months (about 2.5°C rise) is similar to the magnitude of changes observed in the other catchments. This suggests that soil warming could be largest in the spring season in the northern boreal region.

## Discussion

### 6.1 Climate variability and change

Several long-term monitoring studies have shown a possible warmer world toward the end of 20th century [3], [5], [9]. This trend might continue in the future if the current levels of human activities persist. The modelling analyses presented in this study showed that ensemble projections performed better in constraining the uncertainty in future climate change impacts across the climatic gradient of Sweden. This is because no single model can accurately represent the magnitude of change expected in the future considering the level of uncertainty inherent in our current forecasting capability. However, the sign of the changes observed from this study and by others [20], [51] could give some insights on how to devise plausible mitigation strategies for an uncertain future. Although our projected changes on annual scale are large, they fall within the range of values projected by other studies. For example, *Kellomäki et al.* [52] projected an up to 4.5°C rise in air temperature over Finland amidst other projections in Sweden [44], [51]. While annual averages provide a quick overview of climate impacts, they might be uninformative in high latitude boreal catchments unless complemented by seasonal projections. Our seasonal analysis showed that impacts could be more pronounced during winter months and the magnitude of the change in air temperature increases northward. This is because the period with winter air temperatures <0°C was projected to shorten considerably in the northern catchments that are characterized by significant snow cover which may disappear completely in the south. This will have large impacts on ecohydrology and biogeochemical processes including snow accumulation and melting, soil frost [53], ecosystem and forest productivity [19], changes in water quality [5], distribution of tree species and community composition [54] amongst others.

### 6.2 Soil temperature modelling

The extended soil temperature model presented in this study offers improved soil temperature simulations as heat-flow are allowed to influence the soil layer of consideration from below. This is very important in understanding other biogeochemical processes as soil temperatures are becoming represented in other environmental source assessment models [36], [42], [55]. Recently, *Winterdahl et al.* [56] observed improved simulations of stream dissolved organic carbon concentration when soil temperature was integrated to the riparian processes. This showed a need for proper accounting for soil temperature in biogeochemical studies [57] and to further constrain uncertainty in biogeochemical models [34], [35] for credible assessments of possible futures.

The deepest soil layers consistently showed less variability than the topmost layers. This is explainable as topmost layer can be influenced by fluctuations in daily air temperature. This is particularly notable in Kindla and Gårdsjön where bedrock outcrops [38] can conduct additional heat from the atmosphere to the soil. In general, Gårdsjön has shallower soils that can make the top two layers more sensitive to daily air temperature variability. However, the large variation in the earlier observations at Aneboda could not be explained in terms of soil physical properties related to the proportion of soil wetness (17% in Aneboda; 24% in Kindla). Southern Sweden was hit by a severe storm in 2005 followed by pest infestation [38] that destroyed some forest vegetation (more severe in Aneboda than Gårdsjön). However, no generalization could be made on the impact of the storm in Aneboda or influence of soil heterogeneity in each catchment as soil temperature measurements were not made throughout the landscapes.

The soil temperature model presented here successfully captured the inter-annual and seasonal dynamics of soil temperature in each catchment. The high NS values for model calibration are explainable as soil temperature is less variable unlike air temperature noted above. Though high summer and low winter soil temperatures were well simulated, the model slightly underestimated soil temperature in some years especially in northernmost catchment. This was particularly notable during autumn cooling and spring warming. Gammtratten was also the study site where the influence of snow cover change was clearly visible. This is not surprising in this region as similar observations were also reported in neighboring Finnish catchments [33].

General uncertainty analysis showed that the model showed equifinality [50] due to strong correlation between parameters. However, the parameter *C_S,ICE_* appeared to be highly insensitive. This observation is not surprising since *C_S,ICE_* only comes into play when soil temperatures drop below zero. This argument also holds for parameter *f_S_*, which only affects NS when there is snow. Calibrating only for the winter would reveal more about the true sensitivity of the mentioned parameters and this approach would be attempted in future work. However, sensitivity of K_T_ showed the importance of soil thermal conductivity and that an assumption of uniformity across soil profiles or catchments should not be made. This is important as *Euskirchen et al.* [11] clearly showed the significance of change in soil thermal conductivity on growing season length and forest productivity across boreal ecosystems.

### 6.3 Ensemble projections

**Figure 7:**
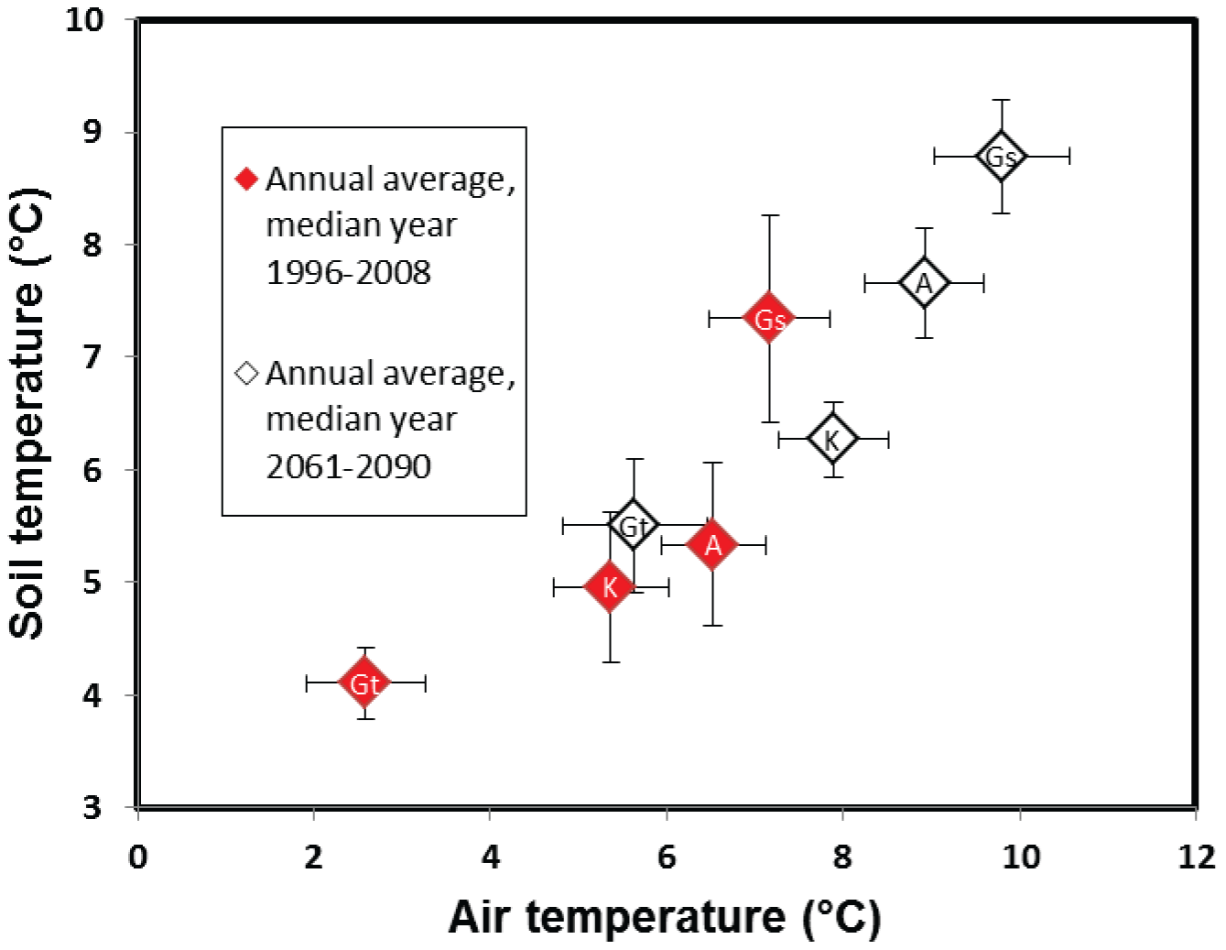
Summary of projected change in long term annual soil temperature using the RCM ensemble median (white *diamonds*) for Aneboda (*A*), Gårdsjön (*Gs*), Kindla (*K*) and Gammtratten (*Gt*) versus corresponding observed median soil temperatures for the test period (*red diamonds*). *Error bars* represent corresponding annual standard deviations.

This study shows that the relationship between air and soil temperature responses is not linear and that complex, climate-related feedbacks will increase in northern boreal biomes. Our projected range of future soil temperature responses is consistent with other studies including a recent study [58] which projected an up to 3.3°C rise in soil temperature in southern Quebec forest catchments in Canada. *Zhang et al.* [9] previously used the process-based NEST model across a climatic gradient in Canada and showed that soil temperature (at 20 cm depth) increased by 0.6°C for 1°C rise in air temperature. They attributed the difference to the effect of more insulation from snow cover in winter in high latitude catchments. Our study also indicated that air temperature could increase more rapidly than soil temperature across the climatic gradient of Sweden. This could make future soil/air temperature in northernmost Gammtratten shift towards present day conditions in the south by the end of the century (Figure 7). One possible implication of the results presented here is a shift in biomes from middle-boreal that characterize the northernmost Gammtratten catchment to southern- boreal or in the worst case, boreo-nemoral that characterize southern Sweden.

The large soil temperature changes and possibility of lesser snowfall in the southern catchments due to warmer winter will have biogeochemical implications for peaty soils. Increased winter soil wetness and less frost could reduce the carrying capacity of the soil for winter forestry operations [52]. Though drier soils could support deep winter soil frost under warmer conditions [26], [53] but possible persistent soil frost in the north could also be of ecological concern. For example, our results showed possibility of minimal winter soil temperature changes in Gammtratten catchment. Although this remains around the freezing point (Figure 5), there is a possibility of slightly colder soils. This borderline winter soil temperature responses around the freezing point in Gammtratten is not surprising as *Campbell et al.* [59] recently reported similar results from the Hubbard Brook Experimental Forest in the United States. Their SHAW model similarly showed some biases in partitioning precipitation to either rainfall or snowfall around the freezing point. They suggested that precipitation would be a less important factor influencing snowpack and soil frost in the winter in the future. This partly justifies our lack of snowfall data to constrain this uncertainty in our model but the tendency of decreasing winter soil temperature at Gammtratten is not unambiguous as whether it would result from less or more snow cover. Several studies have also shown possibility of colder winter soils as the future gets warmer [22], [3]. Therefore, warmer air temperature can be a limiting factor for deep soil frost as length of frost days could be concurrently reduced. Decreasing frost days are evident at Gammtratten where the model showed possible largest increase in soil temperature in the spring. This is an indication of a faster snowmelt regime and as a result could lead to extended growing season in the future. Recent analysis of historical data by [5] had shown already increasing trends in the growing season length in this region

*Strömgren and Linder* [19] conducted a soil warming experiment and showed that heated plots also had less snow cover and favored more stem volume production. This was due to possible extension of growing season [11] and ambient environment that favors more nutrient availability [14]. *Bergh and Linder* [60] also conducted a proxy study of future climate effects by burying heating cables in the soil. They showed that snowmelt and soil thawing occurred earlier in heated than unheated plots. Several other snow manipulation experiments have also shown that the persistent deep soil frost could increase organic matter leaching [10] and lower rates of winter respiration [13]. A drastic reduction in cellulose decomposition (up to 46%) was observed in snow removal experiment in northern boreal forest in Sweden [29].

To further test the internal working process of the model presented in this study, application of the model to catchments with both soil temperature and snow data would be worthwhile to constrain the uncertainty in model structure and future. Comparing the soil temperature measurements from the same depth in the soil profiles could give more credible comparison between sites to evaluate whether parameter behaviors are due to soil type or changing soil properties down the profile. While this is important to constrain the model uncertainty, it might be difficult to attain. One recent study [61] showed that soil properties can significantly affect thermal distribution in soil profiles such that direct comparison could be difficult even if the measurements were taken at the same depth in each catchment. Future work should also consider using only winter soil/air temperatures data for parameter estimation when simulating winter temperatures. The overall conclusion of this study showed that the effects of climate change on soil temperature responses in snow dominated region is complex. General assumptions of soil temperature dynamics based on future air temperature change alone are not enough and should be avoided in high latitude ecosystems.
